# CoGAPS matrix factorization algorithm identifies transcriptional changes in AP-2alpha target genes in feedback from therapeutic inhibition of the EGFR network

**DOI:** 10.18632/oncotarget.12075

**Published:** 2016-09-16

**Authors:** Elana J. Fertig, Hiroyuki Ozawa, Manjusha Thakar, Jason D. Howard, Luciane T. Kagohara, Gabriel Krigsfeld, Ruchira S. Ranaweera, Robert M. Hughes, Jimena Perez, Siân Jones, Alexander V. Favorov, Jacob Carey, Genevieve Stein-O'Brien, Daria A. Gaykalova, Michael F. Ochs, Christine H. Chung

**Affiliations:** ^1^ Department of Oncology, Sidney Kimmel Comprehensive Cancer Center, Johns Hopkins University, Baltimore, MD, USA; ^2^ Department of Otorhinolaryngology-Head and Neck Surgery, Keio University School of Medicine, Tokyo, Japan; ^3^ Department of Head and Neck-Endocrine Oncology, Moffitt Cancer Center, Tampa, FL, USA; ^4^ Personal Genome Diagnostics, Baltimore, MD, USA; ^5^ Vavilov Institute of General Genetics, Moscow, Russia; ^6^ Research Institute for Genetics and Selection of Industrial Microorganisms, Moscow, Russia; ^7^ Department of Biostatistics, Bloomberg School of Public Health, Johns Hopkins University, Baltimore, MD, USA; ^8^ Institute of Genetic Medicine, Johns Hopkins University, Baltimore, MD, USA; ^9^ Lieber Institute for Brain Development, Baltimore, MD, USA; ^10^ Department of Otolaryngology-Head and Neck Surgery, Johns Hopkins University School of Medicine, Baltimore, MD, USA; ^11^ Department of Mathematics and Statistics, The College of New Jersey, Ewing Township, NJ, USA

**Keywords:** EGFR, targeted therapeutics, cell signaling, genomics, crosstalk

## Abstract

Patients with oncogene driven tumors are treated with targeted therapeutics including EGFR inhibitors. Genomic data from The Cancer Genome Atlas (TCGA) demonstrates molecular alterations to EGFR, MAPK, and PI3K pathways in previously untreated tumors. Therefore, this study uses bioinformatics algorithms to delineate interactions resulting from EGFR inhibitor use in cancer cells with these genetic alterations. We modify the HaCaT keratinocyte cell line model to simulate cancer cells with constitutive activation of EGFR, HRAS, and PI3K in a controlled genetic background. We then measure gene expression after treating modified HaCaT cells with gefitinib, afatinib, and cetuximab. The CoGAPS algorithm distinguishes a gene expression signature associated with the anticipated silencing of the EGFR network. It also infers a feedback signature with EGFR gene expression itself increasing in cells that are responsive to EGFR inhibitors. This feedback signature has increased expression of several growth factor receptors regulated by the AP-2 family of transcription factors. The gene expression signatures for AP-2alpha are further correlated with sensitivity to cetuximab treatment in HNSCC cell lines and changes in EGFR expression in HNSCC tumors with low *CDKN2A* gene expression. In addition, the AP-2alpha gene expression signatures are also associated with inhibition of MEK, PI3K, and mTOR pathways in the Library of Integrated Network-Based Cellular Signatures (LINCS) data. These results suggest that AP-2 transcription factors are activated as feedback from EGFR network inhibition and may mediate EGFR inhibitor resistance.

## INTRODUCTION

Precision medicine in cancer aims to improve therapeutic outcomes by matching intervention to the genetic alterations observed in individual cancers. For example, over-expression of epidermal growth factor receptor (EGFR) has been associated with poor prognosis in head and neck squamous cell carcinoma (HNSCC) [1, 2]. Cetuximab, a monoclonal antibody inhibiting EGFR, has demonstrated improved survival in HNSCC patients when combined with chemotherapy or radiation, leading to Food and Drug Administration (FDA) approval for these approaches [3–7]. Similar findings have led to the FDA approval and clinical adoption of EGFR inhibitors in other solid tumors [8]. However, both *de novo* and acquired resistance are common [8], making durable clinical responses to EGFR inhibitors rare [6].

Previously, we have published molecular alterations to cellular signaling pathways within the EGFR network associated with *in vitro* cetuximab resistance in HNSCC cells [9, 10]. These signaling changes arise from complex feedback [11] between ligand overexpression and receptor crosstalk [10], changes in miRNA expression [10], DNA methylation [12], and genetic alterations [13]. Molecular mechanisms for therapeutic resistance may be present at the time of treatment, may expand due to clonal selection, be acquired during tumor evolution, or adapt from rapid rewiring of cellular signaling pathways [14]. Furthermore, each individual tumor or each sub-clone comprising that tumor may have unique molecular mechanisms for such therapeutic resistance [15–19].

In this study, we hypothesize that genomic signatures from short-term transcriptional responses to EGFR inhibitors will distinguish signaling processes in sensitive and resistant cells. To test this hypothesis, we treat *in vitro* models of EGFR, MAPK, and PI3K pathway activation in HNSCC [9] with gefitinib, afatinib, and cetuximab. EGFR inhibition is also modeled by knocking-down EGFR expression with siRNA. Gene expression is measured in each of these conditions. We apply the CoGAPS meta-pathway analysis algorithm [20] to delineate genomics signatures for cell-signaling responses to EGFR inhibition with genetic alterations in the EGFR signaling network. This algorithm confirms that signaling in the MAPK pathway remains elevated in cells that are resistant to EGFR inhibitors. It also identifies unexpected transcriptional increases in gene expression of AP-2alpha targets when treating EGFR inhibitor sensitive cells with cetuximab, gefitinib, and afatinib. The AP-2alpha growth factor receptor increases gene expression of several growth factor receptors, and may be a mechanism by which sensitive cells maintain homeostasis in growth factor receptor signaling. Thus, this CoGAPS meta-pathway analysis of short-term gene expression data can detect gene expression signatures that are critical early biomarkers for therapeutic sensitivity to EGFR targeted agents.

## RESULTS

### Genetic alterations to EGFR network signaling proteins are pervasive in cancer subtypes treated with EGFR inhibitors

**Figure 1:**
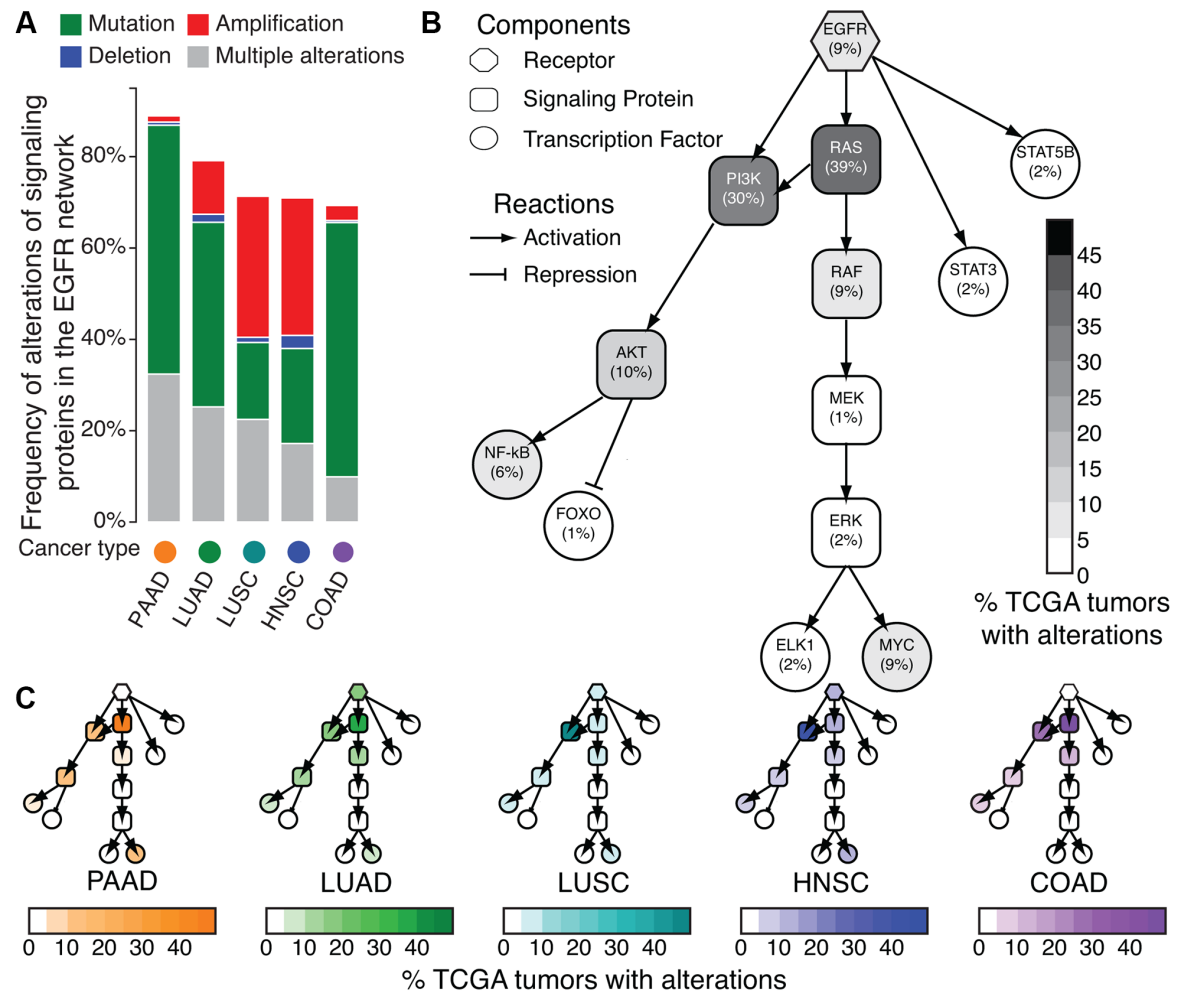
Frequency of DNA alterations to EGFR network signaling proteins in TCGA **A.** Summary of total number of mutations or copy number alterations in the network for pancreatic adenocarcinoma (PAAD), lung adenocarcinoma (LUAD), lung squamous cell carcinoma (LUSC), head and neck squamous cell carcinoma (HNSCC), and colon adenocarcinoma (COAD) tumors in TCGA. **B.** Cell signaling network of EGFR in human tumors. Shading of each node indicates the percentage of samples with alterations in each node of the EGFR cell signaling network across all the TCGA tumor types in **A** according to the color bar. **C.** Cell signaling network of EGFR, with nodes shaded according to percentage of samples with DNA alterations in each tumor type from **A**.

Previously, we described the protein-protein interactions evident in HNSCC-specific EGFR signaling [9] from comprehensive reviews [21, 22]. In this study, we survey the DNA alterations of EGFR signaling proteins in solid tumors represented in The Cancer Genome Atlas (TCGA) and are FDA-approved for EGFR inhibitor treatment [8]: pancreatic adenocarcinoma (PAAD), lung adenocarcinoma (LUAD) [23], lung squamous cell carcinoma (LUSC) [24], HNSCC [25], and colon adenocarcinoma (COAD) [26]. In these tumors, DNA alterations to the EGFR network are pervasive (Figure 1A).

Alterations to distinct signaling proteins within the EGFR network do not exhibit equivalent impact for EGFR inhibitor therapeutic sensitivity. Therefore, we survey the average frequency of genetic alterations corresponding to each signaling protein in the EGFR network across PAAD, LUAD, LUSC, HNSCC, and COAD tumors in TCGA (Figure 1B). *EGFR* amplifications and mutations occur in only 9% of primary tumors in each subtype, with genetic alterations in the PI3K family (*PIK3CA, PIK3CB, PIK3CD*, or *PIK3CG*) (30%) and in the RAS family (*HRAS, KRAS, or NRAS*) (39%) being most prevalent (Figure 1B). Similar molecular landscapes for the EGFR network are observed for each cancer type (Figure 1C, Supplementary Table S1) with RAS alterations most common in PAAD (86%), LUAD (39%), and COAD (50%) and PI3K alterations most common in LUSC (55%) and HNSCC (41%). Thus, we observe that mutations within *EGFR* and the RAS and PI3K pathways are the most common genetic alterations in tumors currently treated with EGFR inhibitors. Because they are downstream of EGFR in the cell-signaling network, both RAS and PI3K alterations confer resistance to EGFR inhibitors [8, 27]. However, neither their absence nor EGFR expression are sufficient to predict long term therapeutic sensitivity [8]. To better inform treatment selection, it is possible that short term changes in gene expression resulting from therapeutic inhibition will define signaling responses associated with treatment sensitivity or resistance against these commonly altered genetic backgrounds.

### Characterization of modified HaCaT cell models with oncogenic EGFR, MAPK, and PI3K pathway activation

**Figure 2:**
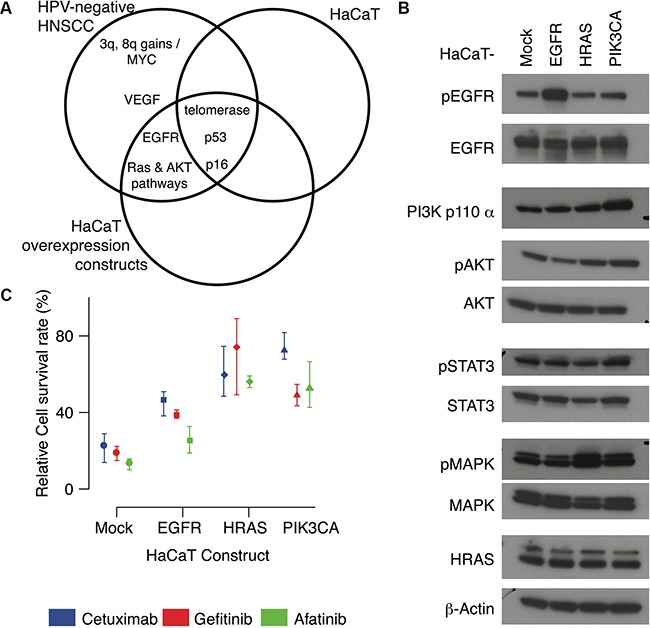
HaCaT overexpression constructs **A.** Venn diagram comparing molecular alterations in HPV-negative HNSCC to the HaCaT keratinocyte cell lines and HaCaT-EGFR, HaCaT-HRAS^V12D^, and HaCaT-PIK3CA^H1047R^ overexpression constructs. **B.** Western blots for each HaCaT cell line. **C.** Total colony area (survival rates) for each HaCaT construct after seven days of treatment with cetuximab (100nM, blue), gefitinib (100nM, red) and afatinib (10nM, green) relative to PBS control, set at 100% of survival. Mean values are indicated as points and error bars represent the maximum and minimum values over three replicates.

The immortalized, but not transformed, HaCaT keratinocyte cell line has been well characterized for the molecular alterations of premalignancy in HPV-negative HNSCC (Figure 2A). Building on the observations of pervasive *EGFR* and RAS/PI3K alterations in TCGA tumors, we introduce overexpression of wild-type *EGFR* overexpression, *HRAS^V12D^*, and *PIK3CA^H1047R^* activating constructs to model the acquisition of these constitutively active alterations during carcinogenesis (Figure 2A). These modified HaCaT cells with activation of specific signaling pathways enable us to delineate signaling responses to molecular or pharmacological perturbation across an isogenic background. RT-PCR confirms *EGFR* mRNA and *HRAS* mRNA are overexpressed in HaCaT-EGFR and HaCaT-HRAS^V12D^ cells with respect to control (HaCaT-Mock) (Supplementary Figure S1). Western blot analysis with phospho-specific antibodies further validates the impact of each alteration on cell signaling within the EGFR network (Figure 2B, Supplementary Figure S2). For example, HaCaT-PIK3CA*^H1047R^* demonstrates enhanced downstream phospho-AKT and phospho-STAT3 levels. Furthermore, HaCaT-EGFR also demonstrates increased phospho-EGFR signaling. Additionally, HaCaT-HRAS*^V12D^* exhibits an expected increase in phospho-MAPK levels (Figure 2B). Therefore, these HaCaT *in vitro* models exhibit the predicted signaling alterations expected by EGFR, RAS, and PI3K pathway activation without the complexity introduced by the broader genetic heterogeneity evident within the landscape of cancer cell lines or human tumors.

After validating each modification, we inhibit EGFR in the HaCaT cell lines with three targeted agents with unique mechanisms of action: cetuximab, gefitinib and afatinib. Cetuximab is a monoclonal antibody specific for the extracellular portion of EGFR, thus it functions to prevent receptor activation by blocking ligand binding. Both gefitinib and afatinib are small molecule tyrosine kinase inhibitors that prevent intracellular activation of the receptor. While cetuximab and gefitinib are selective inhibitors of EGFR, afatinib is a pan-HER family inhibitor, potentially preventing further downstream activation mediated through receptor crosstalk among the HER family members. Equivalent numbers of cells were cultured in Matrigel colony formation assays and treated with standard concentrations of gefitinib (100nM), cetuximab (100nM), or afatinib (10nM). After 7 days, total colony area of the treated cells is compared to that of the untreated control in order to calculate the relative cell survival. Lower relative survival rates correspond to greater therapeutic sensitivity, and higher survival rates to therapeutic resistance. At this dosage, survival rates are comparable for gefitinib and cetuximab, and lower in afatinib treatment for each of the modified HaCaT cells (Figure 2C, Supplementary Figure S3). Each oncogenic modification leads to increased cell survival following EGFR inhibition compared to HaCaT-Mock. Only afatinib decreases cell survival in HaCaT-EGFR to a similar extent observed in HaCaT-Mock. Cell survival is consistently higher in both HaCaT-HRAS^V12D^ and HaCaT-PIK3CA^H1047R^ for all three pharmacological agents relative to PBS controls (Figure 2C). The relative survival rates for gefitinib and afatinib are comparable and lower than the corresponding survival rate for cetuximab in the HaCaT-PIK3CA^H1047R^ cells.

### Transcriptional changes elicited by EGFR inhibition distinguish therapeutic sensitivity in modified HaCaT cells with genomic alterations in the EGFR network

We also measure gene expression profiles of the modified HaCaT cell lines before and after 24 hour treatment with EGFR inhibitors (100 nM cetuximab, 100 nM gefitinib, and 10 nM afatinib). Hierarchical clustering identifies four dominant clusters: (1) treated and untreated HaCaT-HRAS^V12D^ cells, (2) untreated HaCaT-Mock, HaCaT-EGFR, and HaCaT-PIK3CA^H1047R^ cells, (3) treated HaCaT-PIK3CA^H1047R^ cells and cetuximab treated HaCaT-Mock cells, and (4) treated HaCaT-EGFR cells and gefitinib or afatinib treated HaCaT-mock cells (Supplementary Figure S4). We note there is substantial overlap between the genes in the third and fourth clusters, with the separation in hierarchical clustering resulting from larger fold changes of gene expression after treatment in HaCaT-Mock and HaCaT-EGFR cells relative to HaCaT-PIK3CA^H1047R^ cells.

**Figure 3:**
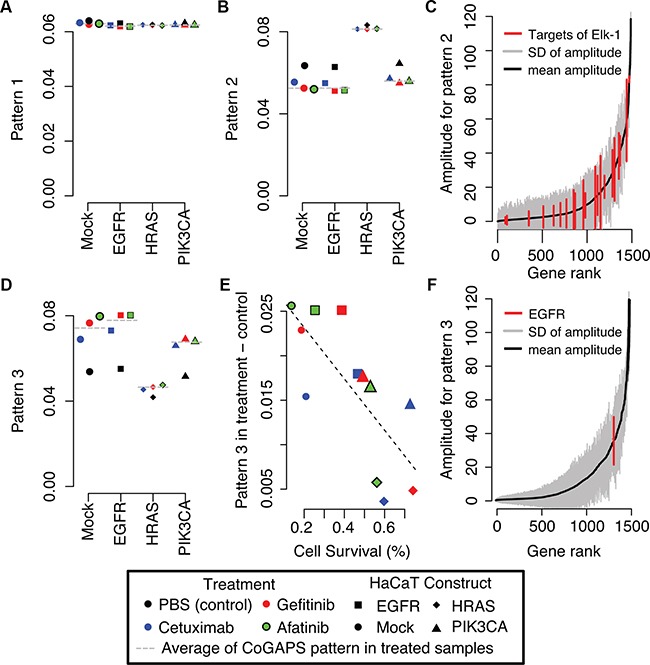
CoGAPS meta-pathways and activity patterns in HaCaT constructs **A.** First CoGAPS pattern capturing genes with limited expression changes in HaCaT constructs or treatment with EGFR inhibitors. **B.** Second CoGAPS pattern with increased gene expression in HaCaT-HRAS^V12D^ samples and decreased gene expression in samples that were treated with EGFR inhibitors. **C.** Magnitude of the corresponding gene associations (amplitude) in all genes (grey) for pattern 2 relative to genes that are targets of transcription factor Elk-1 (red) that is activated by the MAPK pathway. **D.** Third CoGAPS pattern with decreased gene expression in HaCaT-HRAS^V12D^ samples and increased gene expression in samples that were treated with EGFR inhibitors. In **A**, **B**, and **D**, each pattern CoGAPS has a magnitude that is scaled to sum to one across all samples. As a result, samples with pattern values close to zero are unassociated with the given gene signature. On the other hand, samples with pattern values close to one are more strongly associated with the gene signature than any other sample in the data. Values in between one and zero quantify the relative gene expression changes among the genetic backgrounds and treatments. **E.** Comparison of the difference in the magnitude of the third CoGAPS pattern in samples treated with EGFR inhibitors relative to untreated samples to relative cell survival rates after treatment with EGFR inhibitors in each HaCaT construct. **F.** Magnitude of the corresponding gene associations (amplitude) in all genes (grey) for pattern 3 relative to *EGFR* (red).

In addition to hierarchical clustering, we apply the CoGAPS meta-pathway analysis algorithm [28] to this gene expression data. CoGAPS meta-pathway analysis identifies three dominant patterns in gene expression data (Figure 3). Whereas clustering provides exclusive categorical gene and group assignments, CoGAPS infers gene expression signatures with continuous values for each gene enabling individual genes to be associated with multiple pathways. This analysis assigns each sample a continuous value for the association of each gene signature with that sample, called CoGAPS patterns. For this dataset, the first CoGAPS pattern is constant across all cell types and treatments, reflecting constant gene expression values in all conditions (Figure 3A). The second pattern has the highest magnitude in HaCaT-HRAS^V12D^ cells, but simultaneously decreases in all HaCaT cells after EGFR inhibition (Figure 3B). The overexpression of these genes due to the mutant HRAS and their subsequent decrease after treatment was proportional to the relative cell survival of each modified HaCaT cell line (correlation coefficient of 0.72 and p-value of 0.008). Taken together, this pattern is consistent with HRAS and its downstream pathway activation in HaCaT-HRAS^V12D^ cells. It also reflects inhibition of that pathway from treatment with EGFR inhibitors in sensitive cells. The CoGAPS gene set statistic confirms that the gene targets of the transcription factor Elk-1, which we previously confirmed to be a marker of RAS/MAPK signaling [9], are significantly associated with this pattern (Figure 3C, p-value of 0.04).

The third pattern has the lowest magnitude in HaCaT-HRAS^V12D^ cells, but increases in the other modified HaCaT cells after EGFR inhibition (Figure 3D). The observed increase after treatment is highest in HaCaT-Mock and HaCaT-EGFR, consistent with the larger gene expression changes after treatment observed in the clustering analysis (Supplementary Figure S4). In this case, the increase in gene expression changes after treatment is anti-correlated with relative cell survival (Figure 3E, correlation coefficient of −0.75; p-value of 0.005). Thus, the higher the sensitivity of the HaCaT cells to EGFR inhibitors (i.e., lower the relative cell survival), the greater the increase in gene expression changes after treatment. Unexpectedly, gene expression of EGFR itself is associated with this CoGAPS pattern for increased gene expression after EGFR inhibitor treatment (Figure 3F). Moreover, EGFR is significantly overexpressed after treatment with gefitinib (p-value of 5×10^−8^) and afatinib (p-value of 2×10^−6^). A similar trend is observed for cetuximab; however, this difference fails to meet statistical significance (Supplementary Figure S5A; p-value of 0.1).

We also performed siRNA knock-down of EGFR with 86% efficiency at 24 hours in HaCaT mock cells. We compare the resulting gene expression changes after siRNA knock-down relative to siRNA scramble in each of the HaCaT cells (Supplementary Figure S6). In this case, HaCaT EGFR knockdown yields an anticipated and statistically significant decrease in EGFR expression (Supplementary Figure S5B; p-value of 9×10^−6^). This siRNA data suggests that the transcriptional profiling data can effectively detect EGFR inhibition.

### The AP-2alpha family of transcription factors is associated with increased growth factor receptor expression after treatment with EGFR inhibitors

**Figure 4:**
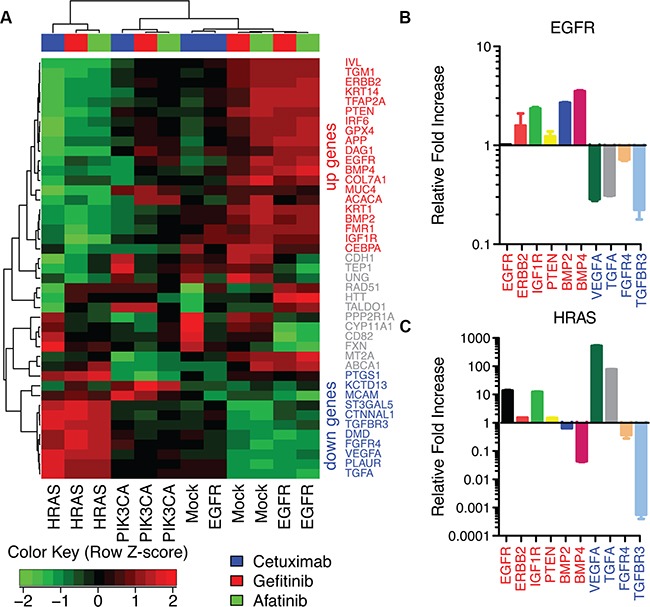
AP-2alpha gene expression signature in HaCaT cells **A.** Heatmap of gene expression changes after treatment with EGFR inhibitors in each HaCaT construct for targets of AP-2alpha. **B.** ΔΔCt values from qRT-PCR for AP-2alpha targets after afatinib treatment relative to PBS control in HaCaT-EGFR cells. **C.** ΔΔCt values from RT-PCR for HaCaT-HRAS^V12D^ cells after afatinib treatment relative to PBS control.

The observed increase in EGFR gene expression in sensitive cell lines after treatment in the third CoGAPS pattern is consistent with a compensatory cellular response resulting from successful EGFR inhibition. Thus, we perform gene set analysis associating the CoGAPS signatures for EGFR expression with transcription factor targets that are annotated in the TRANSFAC database [29] to regulate EGFR expression. This analysis associates AP-2alpha (p-value of 0.03) and AP-2gamma (p-value of 0.02) with EGFR feedback. In addition to EGFR, AP-2alpha and AP-2gamma also regulate the expression of other growth factor receptors. For example, AP-2alpha annotated targets in TRANSFAC include *ERBB2*, *IGF1R*, *PTEN*, *BMP2*, *BMP4*, *VEGFA*, *TGFA*, *FGFR4*, and *TGFBR3*. AP-2gamma targets include *ERBB2* and *TGFBR3*. In addition, AP-2alpha regulates its own expression (*TFAP2A*) and contains the vast majority of annotated targets for AP-2gamma (10 of 14 total target genes, Supplementary Figure S7A). Therefore, we formulate a gene signature for growth factor receptor activation by clustering gene expression changes in AP-2alpha target genes after EGFR inhibitor treatment (Figure 4A). Genes that co-cluster with increased *EGFR* gene expression are labeled as “up” genes in the signature, whereas genes that co-cluster with decreased EGFR gene expression after treatment are labeled as “down” genes in the signature (Figure 4A).

Because AP-2alpha is a transcription factor, its mRNA or protein expression may not directly correlate with its change in activity resulting from EGFR inhibition. Therefore, to confirm AP-2alpha mediated feedback, we measure gene expression of AP-2alpha target genes associated with growth factor receptor signaling which increased (*EGFR*, *ERBB2*, *IGF1R*, *PTEN*, *BMP2*, *BMP4*) or decreased (*VEGFA*, *TGFA*, *FGFR4*, *TGFBR3*) after EGFR inhibition in sensitive cells (HaCaT-EGFR and HaCaT-Mock). Because afatinib treatment has the strongest effect on gene expression, RT-PCR validation of the microarray data is performed only for these samples. Accordingly, RT-PCR analysis confirms consistent gene expression changes after afatinib treatment in all validated genes, excluding *EGFR* in HaCaT-EGFR, a transcript artificially sustained with an exogenous promoter (Figure 4B). This signature is also disrupted after afatinib treatment in HaCaT-HRAS^V12D^ cells (Figure 4C). In this case, expression changes in *BMP2*, *BMP4*, *VEGFA*, and *TGFA* are observed to be in the opposite direction from HaCaT-EGFR after treatment, consistent with the heatmap of AP-2alpha targets in Figure 4A. Also consistent with the clustering analysis, RT-PCR demonstrates modest gene expression changes in HaCaT-Mock (Supplementary Figure S7B) and consistent changes in HaCaT-PIK3CA^H1047R^ (Supplementary Figure S7C) in these target genes after afatinib treatment. In both cases, the gene expression of “down” genes decreased after afatinib treatment similarly to HaCaT-EGFR and in contrast to HaCaT-HRAS^V12D^.

### Gene expression changes from cetuximab treatment in AP-2alpha target genes are associated with therapeutic sensitivity in HPV-negative HNSCC cell lines

**Figure 5:**
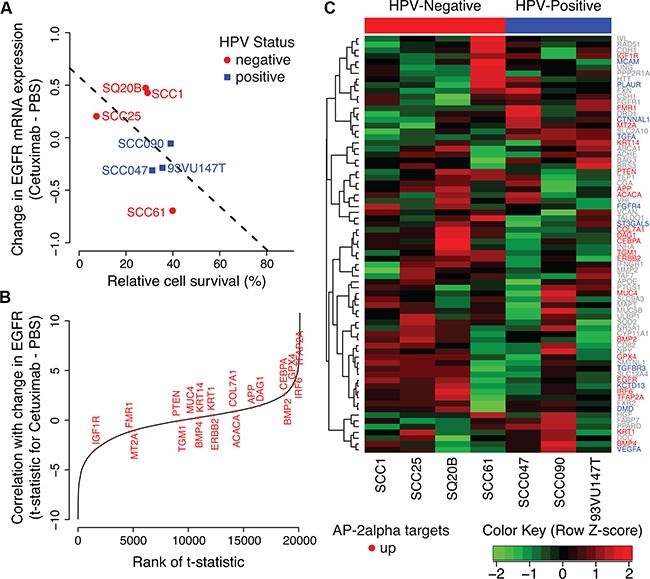
AP-2alpha gene expression signature in HNSCC cell lines **A.** Change in *EGFR* expression after cetuximab treatment relative to PBS control in a panel of HPV-positive (blue) and HPV-negative (red) HNSCC cell lines. **B.** Relative ranking of “up” genes in the AP-2alpha gene expression signature among correlation statistics for changes in gene expression after cetuximab treatment in each gene with the changes in *EGFR* expression. **C.** Heatmap of difference in gene expression between cetuximab treated and untreated HNSCC cells in each AP-2alpha target genes. HNSCC cell lines are ordered according to HPV-status (negative, red and then positive, blue) and then relative cell survival after cetuximab treatment.

We perform further analysis of gene expression data for a panel of HNSCC cell lines treated with 100 nM of cetuximab for 24 hours to confirm our findings in the HaCaT model system in HNSCC cells. Similar to the HaCaT cells, HPV-negative HNSCC cell lines (Figure 5A, Supplementary Table S2) without alterations in the EGFR network (UMSCC1, personal communication with the the Carey and Brenner Labs, publication in process; SCC25, Dataset 1 and [30]) or *EGFR* amplification (SQ20B, Dataset 2) have lower cell survival with cetuximab treatment than cells with *PIK3CA* mutation (SCC61, [31]). Little variation is observed in HPV-positive HNSCC cell lines. We note that this includes a cell line an HRAS mutation (93VU147T, [30]), although this variant is not an activating hotspot mutation. The SCC47 cell line has a 3′ UTR variant in *KRAS* and is sensitive to cetuximab, consistent with observations in human HNSCC for this variant [32].

Also consistent with our observation in the HaCaT model, changes in EGFR mRNA expression in cetuximab treated HNSCC cells relative to control samples are associated with higher cell survival (p-value for one-sided Pearson correlation test of 0.11, Figure 5A). The change in gene expression in *TFAP2A* after cetuximab treatment is significantly associated with the observed change in EGFR expression in all HNSCC cell lines (unadjusted LIMMA p-value of 0.001, Figure 5B). In addition, the “up” genes from the AP-2alpha signature are significantly correlated with the change to EGFR expression in all HNSCC cell lines (one-sided Wilcoxon gene set p-value of 0.02, Figure 5B). However, apparent in the gene expression profiles of the panel of HNSCC cells (Figure 5C), the changes in *TFAP2A* gene expression and AP-2alpha “up” target genes are more strongly associated with changes in *EGFR* expression in HPV-negative cells (p-values of 0.002 and 0.04, respectively) than HPV-positive (p-values of 0.79 and 0.15, respectively). Taken together, these results suggest that AP-2alpha is associated with feedback resulting from therapeutic sensitivity in HPV-negative HNSCC cell lines, whereas an alternative molecular mechanism prevents this feedback in HPV-positive HNSCC cell lines.

### AP-2alpha target genes have coordinated gene expression changes from cetuximab treatment in human HNSCC tumors with low CDKN2A expression

**Figure 6:**
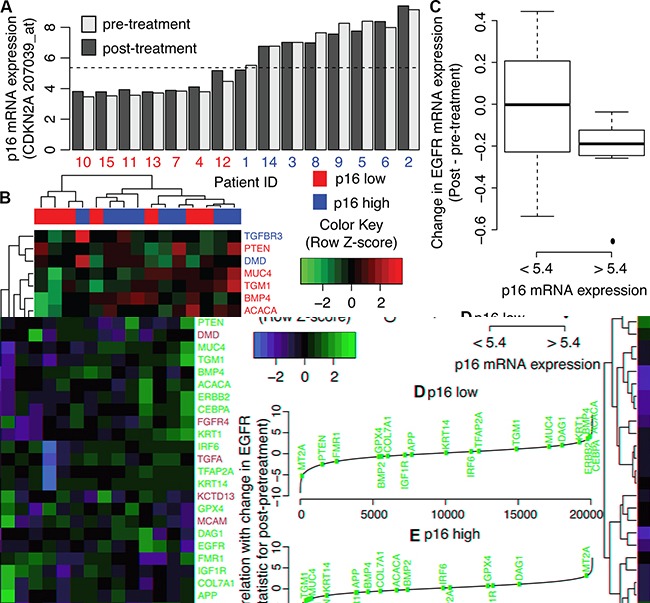
AP-2alpha gene expression signature in human HNSCC pre and post cetuximab treatment biopsies **A.** *CDKN2A* expression in pre- (light grey) and post-treatment (dark grey) biopsies. Values are compared to average *CDKN2A* expression in all samples (dashed-line). This average expression is used as a threshold to label samples as having low (red) or high (blue) *CDKN2A* expression, labeled p16 low and high, respectively. **B.** Heatmap of gene expression changes pre- and post-cetuximab treatment in genes in the **AP-2** alpha signature. **C.** Relative ranking of “up” genes in the AP-2alpha gene expression signature among correlation statistics for changes in gene expression after cetuximab treatment in each gene with the changes in *EGFR* expression in tumors with low *CDKN2A expression*. **D.** As for **C** for tumors with high *CDKN2A expression.*

We perform further analysis of human biopsy samples pre- and post-cetuximab treatment from [33] to assess whether the *AP2-alpha* gene expression signature holds *in vivo*. Whereas the cell lines are from both HPV-negative and HPV-positive cancers, all human biopsy samples are from HPV-negative patients (personal communication with S. Schmitz). Because increased expression of p16, encoded by *CDKN2A*, is often used as a surrogate marker of HPV-positive HNSCC, we evaluate the correlation between *CDKN2A* and *EGFR* expression. We observe a wide range in *CDKN2A* gene expression in these samples that remains consistent between the pre and post treatment biopsies (Figure 6A). Gene expression changes after cetuximab treatment in AP-2alpha target genes cluster according to *CDKN2A* expression (Figure 6B). Consistent with our observation in HPV-negative HNSCC cell lines, changes to *EGFR* expression after cetuximab treatment have greater variability in tumors with low *CDKN2A* expression than high expression (Figure 6C). Expression changes in the “up” genes from the AP-2 alpha signature are more associated with changes in EGFR expression in the tumors with low *CDKN2A* expression than high expression (one-sided Wilcoxon gene set p-value of 0.13, Figure 6D and one-sided Wilcoxon gene set p-value of 1.0, Figure 6E, respectively).

### Expression changes in AP-2alpha target genes are associated with therapeutic inhibition of signaling proteins downstream of EGFR

We perform further analysis of gene expression data from a panel of 9 cancer cell lines from diverse tumor types (Supplementary Table S3 : HA1E, HCC515, A375, A549, HEPG2, HT29, MCF7 PC3, and VCAP) treated with 3,096 therapeutic inhibitors in the Library of Integrated Network-Based Cellular Signatures (LINCS) Program to determine whether the AP-2alpha gene expression signature is also associated with therapeutic response in other cancer types and to other targeted agents. Specifically, LINCS queries the association of changes in gene expression for each of the cell lines with the set of “up” and “down” AP-2alpha targets to rank the association of each therapeutic agent with that signature [34]. LINCS results for all therapeutics are provided in Dataset 3, and summarized for therapeutics that are significantly associated with the AP-2alpha signature in at least four cell lines in Supplementary Table S4.

**Figure 7:**
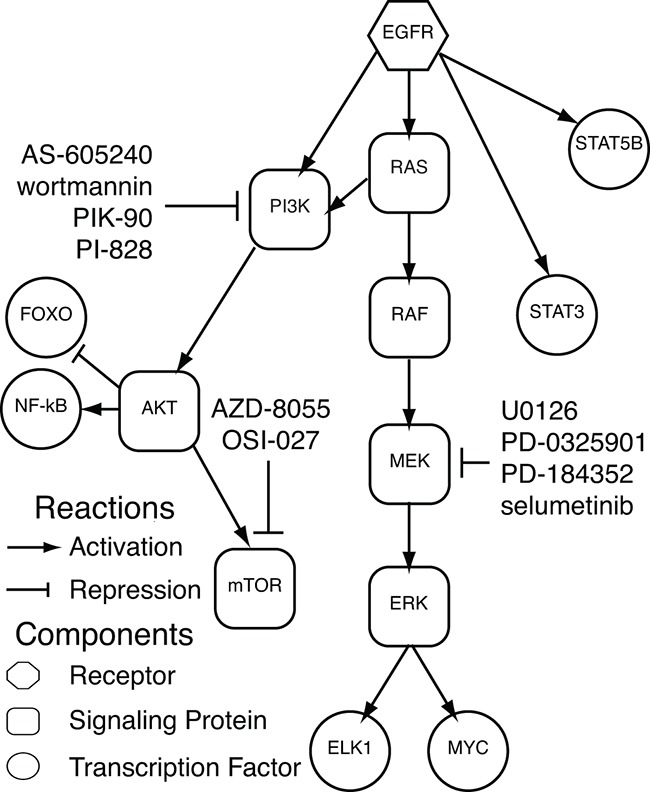
Significantly enriched therapeutic inhibitors in LINCS EGFR cell signaling network including pertubagens from LINCS that inhibit signaling nodes in the EGFR network and are significantly associated with the AP-2alpha gene expression signature.

Similar to our gene expression data from the HaCaT cells, the AP-2alpha signature has mixed association with EGFR inhibitors for distinct cell lines. Specifically, our AP-2alpha signature is positively enriched in 5 of 8 cell lines tested for gefitinib and 5 of 9 cell lines tested for afatinib in LINCS (Dataset 3, Supplementary Table S4). An additional 24 therapeutic agents tested in the LINCS panel are also significantly associated with the AP-2alpha signature with 18 positive and 6 negative correlations. Overall, 10 of the 18 positively correlated agents inhibit signaling proteins downstream of EGFR (MEK, AKT, and mTOR; Figure 7 and Supplementary Table S4). The remaining compounds include IGF1R inhibitors (10-DEBC and BMS-536924), VEGF/VEGFR inhibitors (tivozanib and sunitinib), and a pan-aurora kinase inhibitor (tozasertib). With the exception of PP-110, all of these inhibitors that are associated with overexpression of AP-2alpha targets inhibit signaling proteins downstream of EGFR or growth factor receptors that also activate the signaling proteins in the EGFR network. These LINCS data further suggest that AP-2alpha is associated with a feedback response from effective inhibition of signaling pathways downstream of EGFR.

## DISCUSSION

In this study, we survey the genomic landscape of the EGFR network among cancers in which EGFR inhibitors are often effective [8]. Within the TCGA genomics data for pancreatic, lung [23, 24], head and neck [25], and colon tumors [26], mutations or copy number alterations of EGFR network genes are most pervasive in *EGFR, RAS*, and *PI3K*. Therefore, this study creates a cell line model of EGFR overexpression, mutant HRAS^V12D^, and mutant PIK3CA^H1047R^ in a controlled genetic background. In this model system, activation of the RAS pathway by mutant HRAS^V12D^ and activation of the PI3K pathway by mutant PIK3CA^H1047R^ reduces sensitivity to EGFR inhibitors compared to EGFR overexpression.

Hierarchical clustering analyses of gene expression data in this study determine that gene expression profiles for HaCaT-HRAS^V12D^ cells are less correlated to the gene expression profiles of other modified HaCaT cells. However, these analyses cannot further distinguish gene expression differences between untreated HaCaT-Mock, HaCaT-EGFR, or HaCaT-PIK3CA^H1047R^ cells. Nor does the hierarchical clustering analysis distinguish the gene expression changes between three different EGFR inhibitors: cetuximab, gefitinib, or afatinib. In contrast, our previous study showed that gene expression profiling accurately delineated activated signaling pathways in serum starved HaCaT-HRAS^V12D^, HaCaT-mock, and HaCaT-EGFR cells [9]. These data suggest that perturbations stressing cancer cells enhance differences between gene expression profiles of cells that have molecular alterations that impact therapeutic sensitivity. In these cases, predictions of optimal targeted therapeutic selection from pre-treatment gene expression data alone would be expected to have limited specificity. Such limitations of gene expression profiling of pretreatment samples for precision medicine in cancer will remain regardless of advances to computational algorithms [35–38] or increased availability of high-throughput datasets [39, 40]. Post-treatment data are essential to delineate patients that are responsive to a targeted agent and infer alternative therapeutic modalities for those patients who are resistant. For those that are resistant, there has been a growing call for longitudinal genomics data to infer optimal therapeutic strategies that account for post-treatment cell signaling changes [41]. Studies performing genomics profiling of post-treatment biopsies or repeat surgeries [33, 42, 43], circulating tumor cells and DNA [19, 44], and post-mortem samples [45, 46] are emerging in the literature. However, there are numerous technical challenges to these studies in obtaining enough tissues to characterize the genomics changes, accounting for tumor heterogeneity and the microenvironment [42, 47], profiling patients that are responsive to the therapy, and associating post-treatment samples with robust measures of therapeutic sensitivity. While there is no substitute for genomics profiling of human cancer, pre-clinical models such as the HaCaT cells in this study and mathematical models enable querying of post-treatment genomics changes to numerous therapeutic agents [34], outgrowth of all possible clones from heterogeneous tumor populations [48], and delineate therapeutic sensitivity from cellular density and proliferation [49]. Therefore, longitudinal genomics data from pre-clinical models subject to numerous experimental perturbations are essential to develop the systems biology tools to perform personalized therapeutic selection from longitudinal, post-treatment genomics data.

In this study, the CoGAPS non-negative matrix factorization algorithm [28] infers genes associated with EGFR, PI3K, and HRAS pathway activation more accurately than hierarchical clustering of gene expression data from our modified HaCaT model system. For example, clustering analysis associates gene expression changes in one set of genes with the HaCaT-HRAS^V12D^ cells and another set of genes for sensitive cells treated with EGFR inhibitors. On the other hand, the quantitative pattern detection enabled by CoGAPS infers a single gene expression signature for RAS pathway activation in HaCaT-HRAS^V12D^ cells and RAS pathway repression with EGFR inhibitor treatment in sensitive cells. In the CoGAPS analysis, the magnitude of gene expression decrease evident at 24 hours from the CoGAPS pattern is proportional and predictive of measured sensitivity of modified HaCaT cells after 7 days of EGFR inhibitor treatment. This observation is consistent with the association of activating RAS mutations, which are established resistance mechanisms for EGFR inhibitors [8]. Moreover, our previous study associated a gene expression signature derived from HaCaT-HRAS^V12D^ with acquired therapeutic resistance in the HPV-negative HNSCC cell line, UMSCC1 [9], independent of an acquired RAS mutation in this model system. Based on these results, we anticipate that CoGAPS gene expression signatures will allow for the identification and quantification of the cellular processes associated with targeted agent acquired resistance.

CoGAPS analysis of the gene expression data in this study also identifies an unanticipated increase in growth factor receptor expression after treating HaCaT cells sensitive to EGFR inhibitors. Increases in *EGFR* mRNA expression are also correlated with sensitivity to cetuximab in a panel of HNSCC cell lines. This observation suggests that *EGFR* expression increases when cells with an oncogenic dependence on EGFR signaling are treated with EGFR inhibitors. We hypothesize that increase in *EGFR* expression arises from a feedback mechanism, compensating for its suppression by EGFR inhibitors. It is possible this increase in *EGFR* expression results from a growth advantage in individual cells within our model system, with higher growth factor receptor expression rather than pathway activation that results in transcription of alternate growth signals. However, this change is unlikely to be apparent in gene expression data after the 24 hours of treatment in this study. Negative feedback loops are mathematically associated with greater sensitivity to pharmacological targeting of cell signaling cascades [50]. Therefore, we attribute increased growth factor expression to a feedback mechanism that preserves the homeostasis of *EGFR* expression within cells that are dependent on the EGFR pathway. This feedback mechanism is consistent with the rapid rewiring of signaling networks associated with adaptive resistance to EGFR inhibitors in non-small-cell lung cancer [14].

In previous studies, we have found that high throughput genomics analysis of transcription factor targets can distinguish cellular signaling processes in cancer subtypes [51] and therapeutic response of cells from a single genetic background [52]. This current study finds that AP-2alpha gene targets are correlated with changes in *EGFR* expression after treatment with EGFR inhibitors in our model system. We also use the HaCaT gene expression data to define a transcriptional signature of activated and repressed genes by AP-2alpha from the list of target genes annotated in TRANSFAC. Both *EGFR* expression and that of additional target genes in AP-2alpha also increase concurrently in HNSCC cell lines that are sensitive to cetuximab. Whereas the gene expression changes for HaCaT-HRAS dominate the gene expression signature for HaCaT cells, the *PIK3CA* mutant cell line SCC61 dominates the gene expression signature for HNSCC cell lines. Therefore, we hypothesize that AP-2alpha is a common mechanism for feedback from EGFR inhibition in for all human cancers that are sensitive to EGFR inhibitors.

In the panel of HNSCC cell lines, the association of gene expression changes in the AP-2alpha target genes with increased *EGFR* expression in sensitive cells is stronger in HPV-negative than HPV-positive cells. We observe a similar trend in gene expression data from pre- and post-treatment HPV-negative HNSCC tumor biopsies treated with cetuximab in [33]. Although this dataset lacks measurements of cetuximab response in the tumors, changes in *EGFR* expression after treatment are more variable in tumors with low *CDKN2A* expression. These changes are associated with corresponding changes in AP-2alpha target genes in tumors with low *CDKN2A* expression. Because high p16 expression (encoded by *CDKN2A*) expression is a surrogate biomarker for HPV-related HNSCC [53] and associated with prognosis [54], we hypothesize that the lack of AP-2alpha response to EGFR inhibition in HPV-positive HNSCC are due to that fact that HPV-positive tumors may not be dependent on EGFR, but likely driven by viral oncoproteins, E6 and E7.

To test the association of the AP-2alpha gene expression signature with additional human cancers, we queried its association with therapeutic inhibitors in the broader panel of 9 cancer cell lines and 3,096 therapeutic inhibitors in LINCS. Supporting our initial results, the AP-2alpha signature is associated with gene expression changes from gefitinib and afatinib treatments in a subset of cell lines. Moreover, the AP-2alpha signature is also significantly associated with gene expression signatures of therapeutic agents that inhibit signaling proteins within the EGFR network, including PI3K, AKT, mTOR, and Src, in a greater number of the cell lines from LINCS. These data are consistent with feedback from AP-2alpha being initiated when signaling pathways downstream of EGFR are effectively inhibited. Establishing the functional role of AP-2alpha binding and corresponding transcriptional changes of target genes during this feedback response to targeted therapeutic inhibition warrants further study.

This study associates AP-2alpha with increased growth factor receptor expression after treatment with EGFR inhibitors in sensitive cells. We hypothesize that the observed AP-2alpha transcription of growth factor receptors may give rise to subsequent development of acquired therapeutic resistance to EGFR inhibitors. This compensation is analogous to our previous observation that HB-EGF overexpression is associated with cetuximab resistance in HNSCC [10]. Recently, AP-1 has similarly been associated with acquired resistance to cetuximab in HNSCC and in epidermoid carcinomas [55, 56]. Given the similarity of AP-1 and AP-2 DNA binding domains and their recognized DNA sequence homology [57], it is likely both transcription factors can fulfill similar, but unique, functional roles in mediating acquired cetuximab resistance in HNSCC cells with low *CDKN2A* expression and other cancers treated with EGFR inhibitors. Therefore, we hypothesize that combining new therapeutics to target AP-family transcription factors with therapeutics targeting proteins in the EGFR signaling network will mitigate the feedback response that drives acquired resistance.

In summary, matrix factorization algorithms for transcriptional data [58–60] are powerful discovery tools that enable quantification of relative relationships between samples in complex experimental designs including distinct genetic background and therapeutic conditions. Previously, we showed that CoGAPS is a particularly robust matrix factorization algorithm for inference of transcriptional regulatory networks in cancer [61]. In our current study, the CoGAPS matrix factorization analysis identifies AP-2alpha as a key feedback mechanism initiated by EGFR therapeutic inhibition. The role of AP-2alpha in mediating EGFR targeted agent response and subsequent acquired therapeutic resistance warrants further study.

## MATERIALS AND METHODS

### Cell lines and reagents

Cell line authenticity was confirmed using the Short Tandem Repeat (STR) Identifier kit (Applied Biosystems). HaCaT cells were cultured in W489 media consisting of 80% MCDB153 and 20% L15 medium supplemented with 1% FBS and maintained at 37°C in a humidified incubator with 5% CO_2_. All primary and secondary antibodies were purchased from Cell Signaling Technology (Boston, MA) except anti-HRAS antibody (Santa Cruz Biotechnology, Inc.). Cetuximab (Bristol-Myers Squibb, Princeton, NJ) was purchased from Johns Hopkins Pharmacy. Gefitinib was purchased from Tocris Bioscience (Ellisville, MO). Afatinib (BIBW2992) was purchased from Sellekchem. HaCaT, a spontaneously immortalized keratinocyte cell line was purchased from Cell Lines Service Germany. HaCaT cells were treated with cetuximab (100nM) and gefitinib (100nM), as described previously [10, 62], and afatinib (10nM) based on the dose-response curves for HaCaT-Mock cells. HNSCC cells are grown and treated with cetuximab (100 nM), as previously described [10, 62].

### Generation of HaCaT-Mock, HaCaT-EGFR, HaCaT-HRAS^V12D^, and HaCaT-PIK3CA^H1047R^ cells

To establish continuous expression vectors for EGFR, HRAS^V12D^ and PIK3CA^H1047R^, pLenti-CMV puro lentiviral vector was used. pLenti-EGFR was made by Gateway technology (SIGMA) using Ultimate™ ORFCard for Clone ID IOH81788. pLenti-HRAS was purchased from Addgene (Cambridge, MA). pLenti-PIK3CA^H1047R^ was made from JP1520 PIK3CA H1047R HA vector [63] as template. All vectors were confirmed to have correct gene sequence by sequencing. Viral particles were produced by co-transfecting 900 ng of each expression vector, 100 ng of psPAX2 packaging vector, and 1 μg pMD 2G as an envelope vector into HEK-293T cells using 10 μl of Lipofectamine2000 (Invitrogen Life Technologies). Virus culture supernatants were obtained 24-48 hour after transfection. HaCaT cells were exposed to virus-containing media for 24hr and were selected using puromycin (Invitrogen) 5 ug/mlfor for at least 2 weeks. For confirmation of EGFR or HRAS expression, total RNA was extracted from transduced cell line using Qiagen RNeasy Mini kit (Qiagen, Valencia, CA) according to the manufacturer's protocol. EGFR and HRAS over-expression was confirmed using *EGFR* (Hs01076078_m1) and *HRAS* (Hs00978050_g1) primers by Real time PCR (Applied Biosystems/Life Technologies). Data were analyzed as ΔΔC_t_ with respect to *ACTB*.

### Immunoblotting

Protein lysates were prepared as previously described [10]. Lysed protein concentration was measured by the bicinchoninic acid (BCA) method (Thermo Scientific). Proteins from each sample were fractionated by SDS-PAGE and transferred to nitrocellulose membrane. After blocking with blocking buffer (Li-cor Bioscience), the membranes were incubated with primary antibodies overnight at 4°C followed by incubation with HRP-linked secondary antibodies. Protein bands were visualized by chemiluminescence using the ECL Western blotting Detection System (GE Healthcare, Piscataway, NJ, USA). Western blot quantification was performed with Image Studio Lite v.5.x software (Li-cor). Quantification is determined by the number of pixels detected in a delimited area. We manually determined the area to be quantified for each of the proteins expressed by the HaCaT cell line and by each of the mutant variants generated. We used β-Actin pixel count to normalize the data (target protein/β-Actin) and verify if there is gain or loss in expression relative to normal protein levels.

### Colony formation assay

1×10^3^ cells with 100 μl media were seeded to each well of a 96 well dish coated with 45 ul of Matrigel (BD). Drug treatments were added the next day. Media and reagents were replaced every 3 days. Colonies were scanned and analyzed with GelCount™ (Oxford Optronix Ltd; UK) on day 7 after MTT [4mg/ml 3-(4,5-Dimethythiazol-2-yl)-2,5-diphenyltetrazolium bromide, Sigma-Aldrich] staining for 2 hours. The total area of colonies was calculated by average colony area multiplied by colony number. Relative survival is computed as the total colony area for 7 days of treatment relative to PBS control as described previously [62].

### siRNA knockdown

For knockdown of EGFR, Human Kinase EGFR (siRNA1) MISSION® siRNA (SIHK0657; Sigma-Aldrich) was used. Transfection of siRNA was performed using the Lipofectamine 2000 transfection reagent (Invitrogen) following the manufacturer protocol and assayed for silencing 24 and 48 hours after transfection.

### Microarray data preprocessing and analysis

Gene expression was measured in triplicate using Affymetrix hgu133plus2.0 arrays with data collected in three distinct technical batches. We normalized these arrays with the Bioconductor package fRMA version 1.16.0 [64] and applied pSVA [65] to correct for batch effects. Clustering analysis based upon Euclidean distances was applied to identify outlier samples that still do not cluster with replicate samples after batch correction to remove from subsequent analysis. We selected a single probe for each gene by finding the probe with the highest median absolute deviation between experimental conditions in the HaCaT cells relative to the median absolute deviation across replicates of each experimental condition (Dataset 4). Normalized and raw data are available from GEO (HaCaT cells: GSE80667, HNSCC cells: GSE62027, and untreated UMSCC1: GSE21483).

Raw data for human HNSCC tumors pre and post cetuximab from [33] was provided by personal communication with Sandra Schmitz and Jean-Pascal Machiels and normalized with fRMA. These samples were said to be p16 low if the maximum expression value of the probe selected for *CDKN2A* expression (207039_at) is less than the median average expression value for all pre- and post-treatment samples (5.4).

Differential expression statistics comparing changes in gene expression from treatment with EGFR inhibitors were computed with empirical Bayes moderated, t-statistics from a linear model using the R/Bioconductor package LIMMA [66]. Reported p-values are adjusted for false discovery rate using Benjamini-Hotchberg correction [67]. Analyses are organized with the R package ProjectTemplate (version 0.6) and all the R code used for these analyses in this manuscript is available from the GitHub repository EGFRFeedback.

### CoGAPS meta-pathway inference

We applied the CoGAPS meta-pathway analysis algorithm implemented in the CoGAPS [28] Bioconductor package (version 1.99.0) to infer concurrent gene expression changes in multiple experimental conditions. Specifically, CoGAPS is an unsupervised algorithm that factors a matrix of gene expression data (D) with corresponding uncertainty for each matrix element (Σ) as D ~ *N*(AP, Σ), where *N* represents a univariate normal distribution for each matrix element. In this model, A is a matrix whose columns represent the relative expression of genes in each sample, as determined by the corresponding rows of P. The set of genes with non-zero elements in columns of A are called meta-pathways. The magnitude in the corresponding rows of P indicates the relative activity of the inferred meta-pathway in each sample.

The gene expression data matrix D and corresponding uncertainty matrix Σ was estimated as the mean and the standard deviation of replicate samples for the same experimental conditions, respectively. Elements of Σ were set to have a minimum value of 5% of the signal in D. Genes that are not annotated as experimentally validated transcription factor targets in TRANSFAC [29] professional database (version 2014.1) or with log fold change below 0.5 between any experimental conditions were filtered from analysis. CoGAPS was run on this data for 50,000 iterations for a range from two to six dimensions (columns of A and rows of P). The dimensionality of the data was determined from pattern robustness [68].

### Transcription factor enrichment analysis

A z-score was computed for each element of the A matrix as the ratio of its mean value over samples from the CoGAPS MCMC chain to the uncertainty of samples from the chain. Each pattern was associated with transcription factors by comparing the magnitude of values within the corresponding column of the z-scorefor genes selected for CoGAPS analysis and annotated as targets of that transcription factor in TRANSFAC [29] professional database (version 2014.1, Dataset 5) using the CoGAPS gene set statistic [28]. In order to associate further changes in expression of an individual gene to a transcription factor, we computed the Pearson correlation between the row of the z-scores for the amplitude matrix of that gene in all patterns to corresponding z-scores for every other measured gene. We then filtered the list of transcription factors to those that have the reference gene annotated as a target. We applied a Wilcoxon gene set statistic to compare the correlation coefficients for targets of the remaining transcription factors to the remaining set of genes. We excluded the query gene from the list of genes as annotated targets or the null for this gene set analysis.

### AP-2alpha gene expression signature and qRT-PCR validation

We computed gene expression differences between treated and untreated samples relative to control in each HaCaT cell line for targets of AP-2alpha. Clustering analysis was used to define a gene expression signature of AP-2alpha targets that increased in expression after EGFR inhibition in sensitive lines (called “up” genes) and genes with decreased expression after treatment in sensitive lines (called “down” genes). Additional qRT-PCR validation was performed using Taqman probes (Applied Biosystems, Foster City, CA) on AP-2alpha targets for *EGFR* (HS01076078_m1), *ERBB2* (HS01001580_m1), *IGF1R* (HS00609566_m1), *PTEN* (HS02621230_s1), *BMP2* (HS00154192_m1), *BMP4* (HS00370078_m1), *VEGFA* (HS00900055_m1), *TGFA* (HS00608187_m1), *FGFR4* (HS01169908_m1), and *TGFBR3* (HS00234257_m1) and control β*-actin* (HS00357333-g1) as described above.

### TCGA analysis

TCGA level 3 mutation and GISTIC copy number calls for each gene in the EGFR network were obtained from cBioPortal [69] for LUAD, LUSC, PAAD, COAD, and HNSCC (Dataset 6). A sample with a mutation, a GISTIC score of 2 (copy number amplification), or a GISTIC score of −2 (homozygous deletion), was defined as having an alteration in that gene. Alterations were summarized for genes of the same family, so that PI3K alterations reflected alterations in *PIK3CA*, *PIK3CB*, *PIK3CG*, or *PIK3CD*; RAS to *HRAS, KRAS, or NRAS*; AKT to *AKT1*, *AKT2*, or *AKT3*; RAF to *BRAF* or *ARAF*; and NF-KB to *NFKB1*, *NFKB2, RELA*, or *RELB*.

### DNA alterations in cell lines

DNA alterations for genes in the EGFR network in HNSCC cell lines (SQ20B, SCC61, SCC47, SCC90, and 93VU147T) and cell lines from LINCS (A375, A549, HCC515, HEPG2, HT29, MCF7, PC3, and VCAP) were compiled from the Catalogue of Somatic Mutations in Cancer (COSMIC) [30], [31, 32, 39, 70], and whole exome sequencing for SCC1 (personal communication with Carey and Brenner Labs, publication in process) in Supplementary Tables S2 and S3, respectively. In addition, whole exome sequencing was performed on HNSCC cell lines SCC25 (Dataset 1) and SQ20B (Dataset 2) with the following methods. Fastq files containing raw reads for these cell lines are available on SRA (SRP082979).

### Sample preparation of SCC25 and SQ20B for next-generation sequencing

Sample preparation, library construction, exome capture, next-generation sequencing, and bioinformatics analyses of SCC25 and SQ20B cell lines were performed at Personal Genome Diagnostics (Baltimore, MD). In brief, DNA was extracted from cell lines using the QiaAmp DNA Blood kit (Qiagen). Genomic DNA from cell line samples were fragmented and used for genomic library construction. Briefly, 1-2 micrograms (μg) of genomic DNA in 100 microliters (μl) of TE was fragmented in a Covaris sonicator (Covaris) to a size of 150-450bp. To remove fragments smaller than 150bp, DNA was purified using Agencourt AMPure XP beads (Beckman Coulter) in a ratio of 1.0 to 0.9 of PCR product to beads twice and washed using 70% ethanol per the manufacturer's instructions. Purified, fragmented DNA was mixed with 36 μl of H2O, 10 μl of End Repair Reaction Buffer, 5 μl of End Repair Enzyme Mix (cat# E6050, NEB). The 100 μl end-repair mixture was incubated at 20°C for 30 min, and purified using Agencourt AMPure XP beads (Beckman Coulter) in a ratio of 1.0 to 1.25 of PCR product to beads and washed using 70% ethanol per the manufacturer's instructions. To A-tail, 42 μl of end-repaired DNA was mixed with 5 μl of 10X dA Tailing Reaction Buffer and 3 μl of Klenow (exo-)(cat# E6053, NEB). The 50 μl mixture was incubated at 37°C for 30 min and purified using Agencourt AMPure XP beads (Beckman Coulter) in a ratio of 1.0 to 1.0 of PCR product to beads and washed using 70% ethanol per the manufacturer's instructions. For adaptor ligation, 25 μl of A-tailed DNA was mixed with 6.7 μl of H2O, 3.3 μl of PE-adaptor (Illumina), 10 μl of 5X Ligation buffer and 5 μl of Quick T4 DNA ligase (cat# E6056, NEB). The ligation mixture was incubated at 20°C for 15 min and purified using Agencourt AMPure XP beads (Beckman Coulter) in a ratio of 1.0 to 0.95 and 1.0 of PCR product to beads twice and washed using 70% ethanol per the manufacturer's instructions. To obtain an amplified library, twelve PCRs of 25 μl each were set up, each including 15.5 μl of H2O, 5 μl of 5 x Phusion HF buffer, 0.5 μl of a dNTP mix containing 10 mM of each dNTP, 1.25 μl of DMSO, 0.25 μl of Illumina PE primer #1, 0.25 μl of Illumina PE primer #2, 0.25 μl of Hotstart Phusion polymerase, and 2 μl of the DNA. The PCR program used was: 98°C for 2 minutes; 12 cycles of 98°C for 15 seconds, 65°C for 30 seconds, 72°C for 30 seconds; and 72°C for 5 min. DNA was purified using Agencourt AMPure XP beads (Beckman Coulter) in a ratio of 1.0 to 1.0 of PCR product to beads and washed using 70% ethanol per the manufacturer's instructions. Exonic regions were captured in solution using the Agilent SureSelect 50Mb or v.4 kit according to the manufacturer's instructions (Agilent). The captured library was then purified with a Qiagen MinElute column purification kit and eluted in 17 μl of 70°C EB to obtain 15 μl of captured DNA library. The captured DNA library was amplified in the following way: Eight 30uL PCR reactions each containing 19 μl of H2O, 6 μl of 5 x Phusion HF buffer, 0.6 μl of 10 mM dNTP, 1.5 μl of DMSO, 0.30 μl of Illumina PE primer #1, 0.30μl of Illumina PE primer #2, 0.30 μl of Hotstart Phusion polymerase, and 2 μl of captured exome library were set up. The PCR program used was: 98°C for 30 seconds; 14 cycles of 98°C for 10 seconds, 65°C for 30 seconds, 72°C for 30 seconds; and 72°C for 5 min. To purify PCR products, a NucleoSpin Extract II purification kit (Macherey-Nagel) was used following the manufacturer's instructions. Paired-end sequencing, resulting in 100 bases from each end of the fragments was performed using Illumina HiSeq 2000 (SCC25) and 2500 (SQ20B) instrumentation (Illumina).

### Primary processing of next-generation sequencing data and identification of putative somatic mutations

Somatic mutations were identified using VariantDx custom software for identifying mutations in matched tumor and normal samples. Prior to mutation calling, primary processing of sequence data for both tumor and normal samples were performed using Illumina CASAVA software (v1.8), including masking of adapter sequences. Sequence reads were aligned against the human reference genome (version hg18) using ELAND with additional realignment of select regions using the Needleman-Wunsch method. Candidate somatic mutations, consisting of point mutations, insertions, and deletions were then identified using VariantDx across either the whole exome.

VariantDx examines sequence alignments of cell lines samples against an unmatched normal while applying filters to exclude alignment and sequencing artifacts. In brief, an alignment filter was applied to exclude quality failed reads, unpaired reads, and poorly mapped reads in the tumor. A base quality filter was applied to limit inclusion of bases with reported Phred quality scores > 30 for the cell line and > 20 for the normal (http://www.phrap.com/phred/). A mutation in the cell line was identified as a candidate somatic mutation only when (i) distinct paired reads contained the mutation in the cell line; (ii) the number of distinct paired reads containing a particular mutation in the cell line was at least 10% of read pairs, (iii) the mismatched base was not present in >1% of the reads in the unmatched normal, (iv) the alteration was not reported in the 1000 Genome project, present in >1% of the population or listed as Common in dbSNP135 and (v) the position was covered in both the cell line and normal. Mutations arising from misplaced genome alignments, including paralogous sequences, were identified and excluded by searching the reference genome.

Candidate somatic mutations were further filtered based on gene annotation to identify those occurring in protein coding regions. Functional consequences were predicted using snpEff and a custom database of CCDS, RefSeq and Ensembl annotations using the latest transcript versions available on hg18 from UCSC (https://genome.ucsc.edu/). Predictions were ordered to prefer transcripts with canonical start and stop codons and CCDS or Refseq transcripts over Ensembl when available. Finally mutations were filtered to exclude intronic and silent changes, while retaining mutations resulting in missense mutations, nonsense mutations, frameshifts, or splice site alterations. A manual visual inspection step was used to further remove artifactual changes.

### LINCS analysis of AP-2alpha gene expression signature

The LINCS cloud query tool was used to compare the gene signature for AP-2alpha targets to gene expression data from a panel of pertubagens to data from the LINCS Production Phase L1000 data. Output from the LINCS cloud query is provided directly as Dataset 3, with column labels described in [71]. Pertubagens with mean connectivity scores for the AP-2alpha gene signature across four cell lines greater than 95% or below −95% were called as significantly associated with the AP-2alpha gene signature.

## SUPPLEMENTARY MATERIALS TABLES FIGURES














