# Supplementary material for: CoGAPS matrix factorization algorithm identifies transcriptional changes in AP-2alpha target genes in feedback from therapeutic inhibition of the EGFR network
Source: Oncotarget. 2016 Sep 16;7(45):73845–64. doi: 10.18632/oncotarget.12075 (PMC5342018; doi:10.18632/oncotarget.12075)
Supplement: Supplementary file 1 [file oncotarget-07-73845-s001.pdf]

# CoGAPS matrix factorization algorithm identifies transcriptional changes in AP-2alpha target genes in feedback from therapeutic inhibition of the EGFR network

## SUPPLEMENTARY DATA

Connectivity values for the AP-2alpha gene signature are provided in the column corresponding to the name of each queried cell line. Doses and times used to generate the signature are available in Dataset 3.

**Supplementary Dataset 1: Mutation and copy number calls from whole exome sequencing of the SCC25 HNSCC cell line.**

**See Supplementary File 1**

**Supplementary Dataset 2: Mutation and copy number calls from whole exome sequencing of the SQ20B HNSCC cell line.**

**See Supplementary File 2**

**Supplementary Dataset 3: Results from querying the AP-2alpha gene expression signature for HaCaT cells on LINCS cloud.**

**See Supplementary File 3**

**Supplementary Dataset 4: Probe on the hgu133plus2.0 array selected for each gene for analysis.**

**See Supplementary File 4**

**Supplementary Dataset 5: Gene targets of transcription factors from TRANSFAC used for analysis.**

**See Supplementary File 5**

**Supplementary Dataset 6: DNA alterations of genes in the EGFR network for each TCGA LUAD, LUSC, PAAD, HNSCC, and COAD tumor obtained from cBioPortal.**

**See Supplementary File 6**

## SUPPLEMENTARY FIGURES AND TABLES

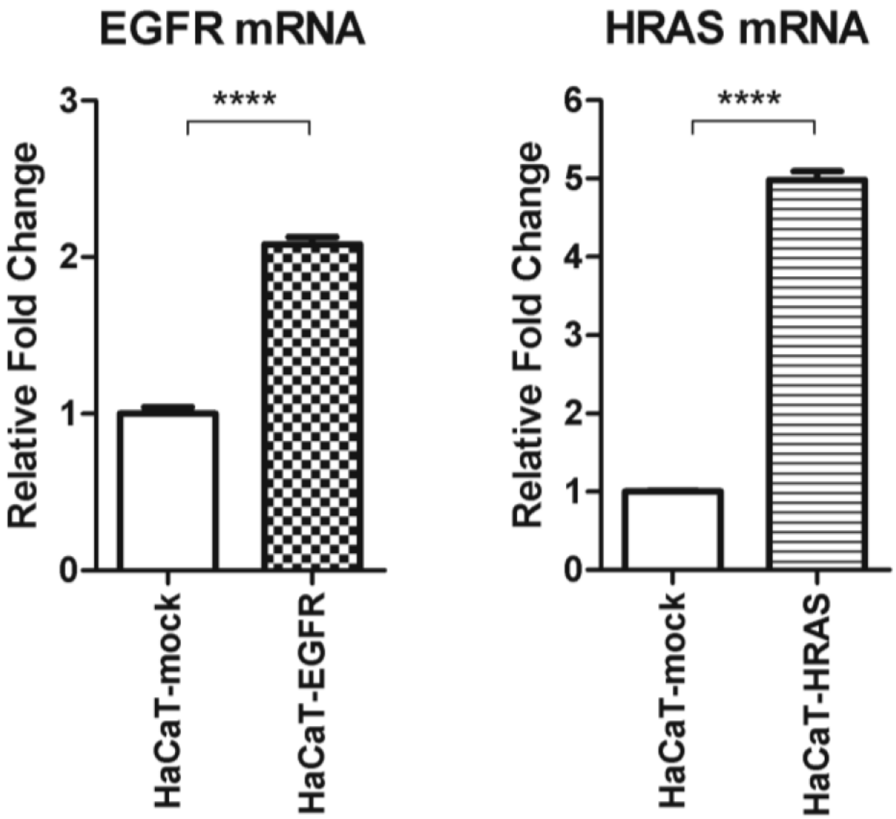


**Supplementary Figure S1: Relative fold change in *EGFR* mRNA for HaCaT-EGFR cells relative to HaCaT-mock and *HRAS* mRNA for HaCaT-mock cells relative to HaCaT-HRAS$^{V12D}$ cells from QRT-PCR.**

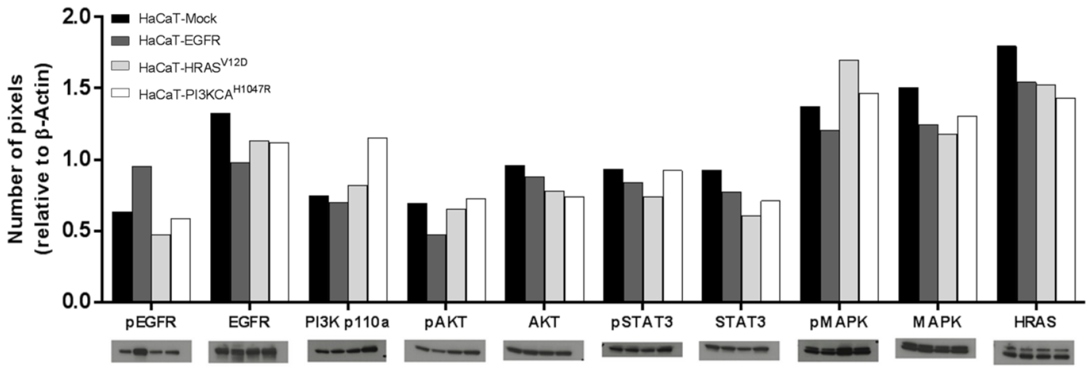


| Cell line | HaCaT-Mock | | HaCaT-EGFR | | HaCaT-HRAS$^{V12D}$ | | HaCaT-PI3KCA$^{H1047R}$ | |
|---|---|---|---|---|---|---|---|---|
| Protein | Pixels | Relative | Pixels | Relative | Pixels | Relative | Pixels | Relative |
| pEGFR | 3,320,000 | 0.63 | 5,890,000 | 0.95 | 2,980,000 | 0.47 | 3,960,000 | 0.59 |
| EGFR | 6,990,000 | 1.33 | 6,060,000 | 0.98 | 7,140,000 | 1.13 | 7,530,000 | 1.12 |
| PI3K p110α | 3,930,000 | 0.75 | 4,310,000 | 0.70 | 5,160,000 | 0.82 | 7,770,000 | 1.15 |
| pAKT | 3,660,000 | 0.70 | 2,940,000 | 0.48 | 4,100,000 | 0.65 | 4,880,000 | 0.73 |
| AKT | 5,060,000 | 0.96 | 5,440,000 | 0.88 | 4,920,000 | 0.78 | 4,970,000 | 0.74 |
| pSTAT3 | 4,910,000 | 0.93 | 5,210,000 | 0.84 | 4,670,000 | 0.74 | 6,220,000 | 0.92 |
| STAT3 | 4,890,000 | 0.93 | 4,790,000 | 0.78 | 3,830,000 | 0.61 | 4,780,000 | 0.71 |
| pMAPK | 7,210,000 | 1.37 | 7,470,000 | 1.21 | 10,700,000 | 1.70 | 9,860,000 | 1.47 |
| MAPK | 7,920,000 | 1.51 | 7,720,000 | 1.25 | 7,430,000 | 1.18 | 8,800,000 | 1.31 |
| HRAS | 9,450,000 | 1.80 | 9,540,000 | 1.54 | 9,590,000 | 1.52 | 9,640,000 | 1.43 |
| β-Actin | 5,260,000 | 1.00 | 6,180,000 | 1.00 | 6,300,000 | 1.00 | 6,730,000 | 1.00 |

**Supplementary Figure S2: Western Blot (WB) quantification.** Bar graphs represent the number of pixels detected by the Image Studio Lite v.5.x software (Li-Cor) relative to β-Actin. Each group of bars represents one protein measured for each cell line and below are the bands from the WB membrane. All quantification data, total number of pixels (Pixels) and relative to β-Actin data (Relative), are shown in the table below the graph.

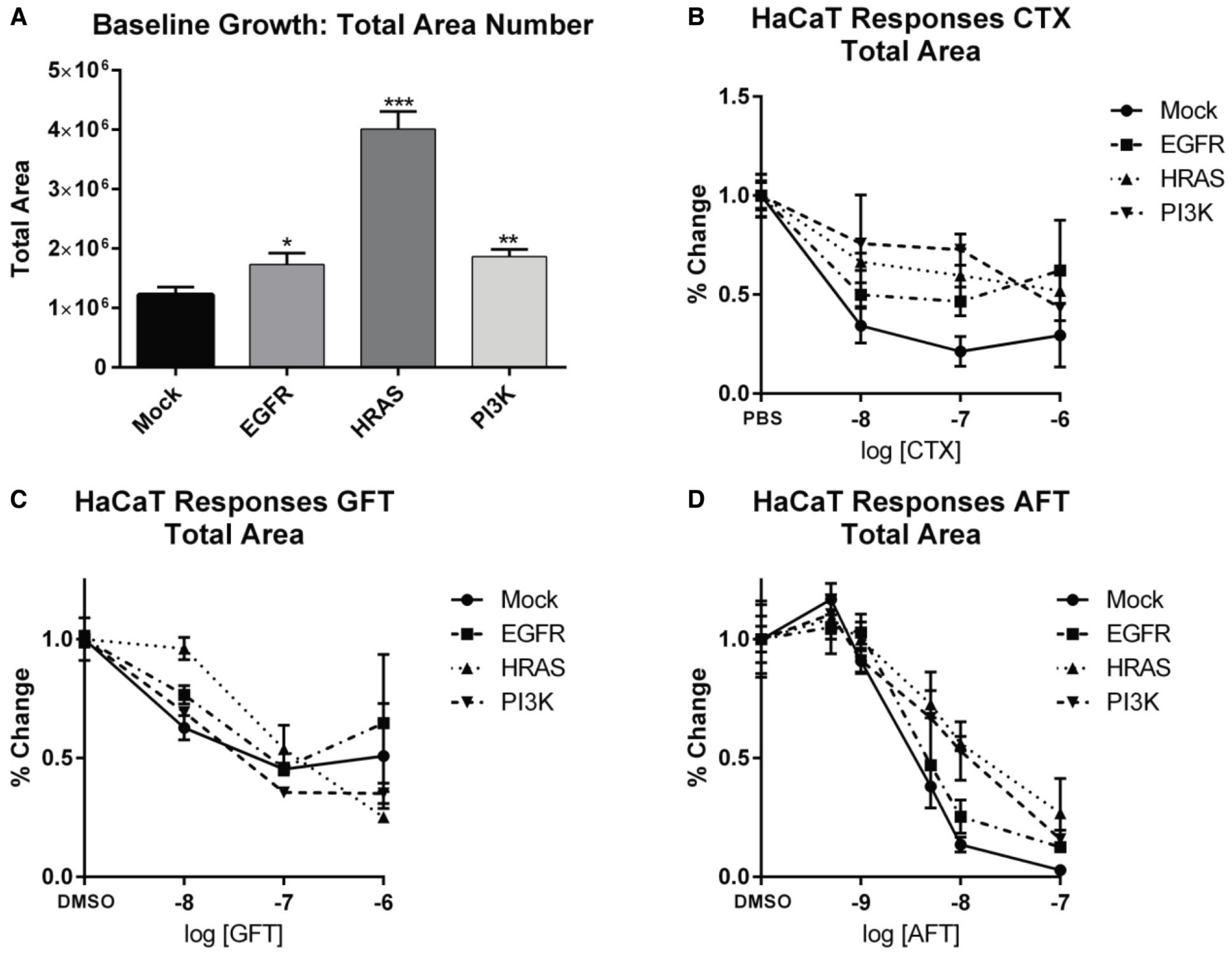


**Supplementary Figure S3: A.** Baseline growth of HaCaT cells at 7 days from total colony area. * indicate statistical significance, where the p-value for the difference in total colony area between HaCaT-EGFR and HaCaT-Mock is 0.02, HaCaT-HRAS$^{V12D}$ and HaCaT-Mock is 0.0001, and HaCaT-PIK3CA$^{H1047R}$ and HaCaT-Mock is 0.003. Dose response curves for cetuximab **B.**, gefitinib **C.**, and afatinib **D.** of total colony of each HaCaT construct relative to baseline, untreated cells at 7 days.

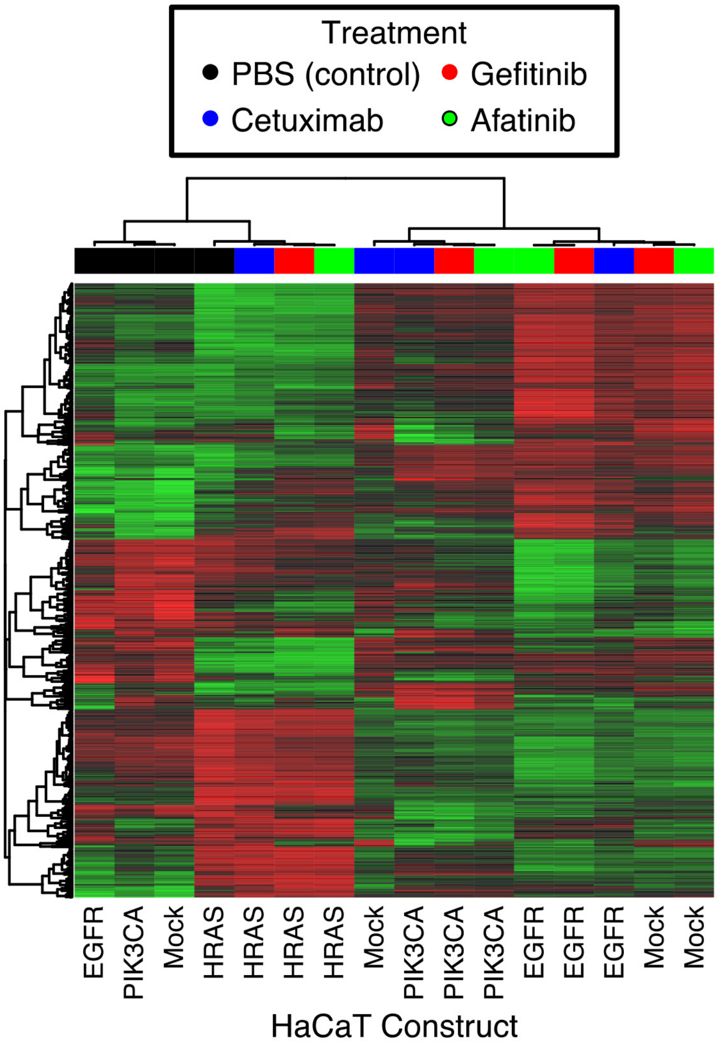


**Supplementary Figure S4: Clustering of gene expression data of HaCaT cells in each treatment condition.** Columns are labeled according to the modification to the HaCaT cells and colored according to the treatment (see color legend).

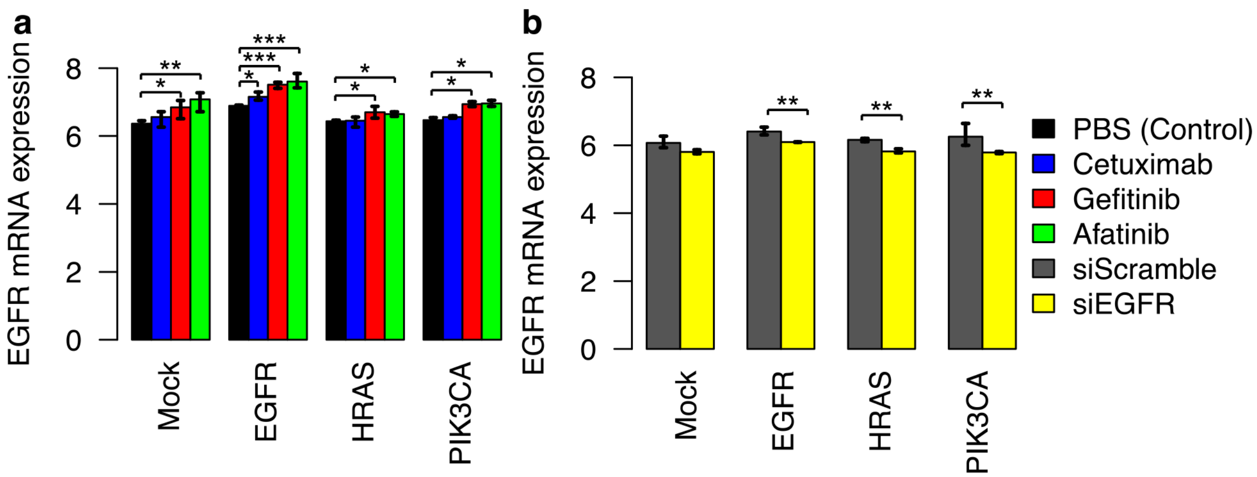


**Supplementary Figure S5: A.** *EGFR* mRNA expression measured with the Affymetrix microarray in each HaCaT construct and treatment condition. **B.** *EGFR* mRNA expression measured with the Affymetrix microarray in each HaCaT construct with siEGFR and scramble siRNA. Statistically significant p-value of treatment relative to PBS control and siEGFR relative to siScramble control are represented by * (< 0.05), ** (< 0.005), and *** (< 0.0005).

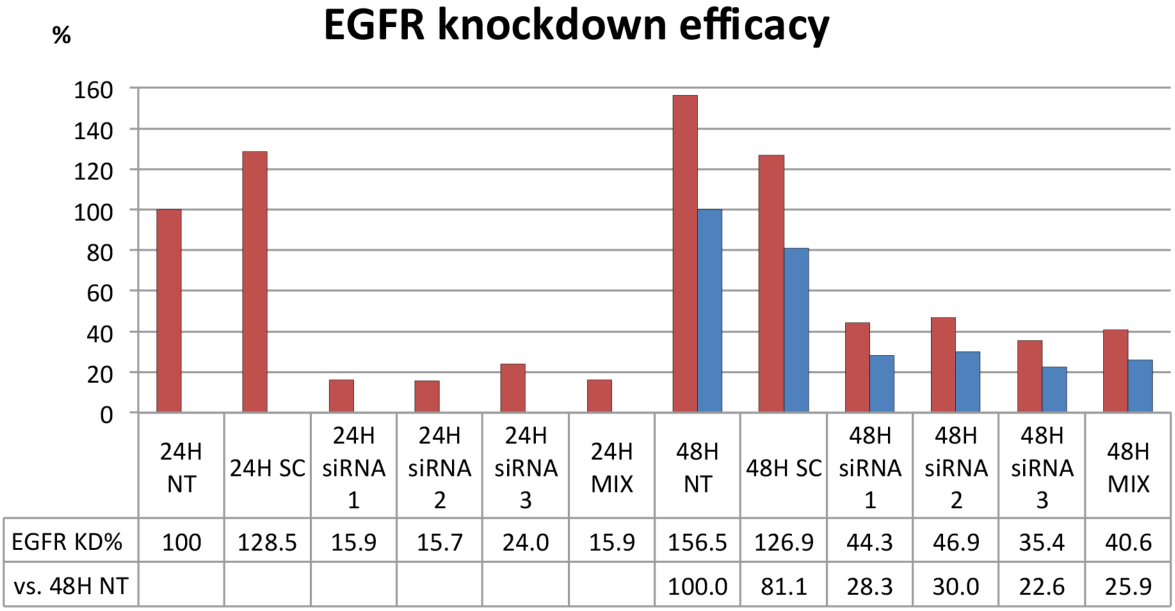


| | 24H NT | 24H SC | 24H siRNA 1 | 24H siRNA 2 | 24H siRNA 3 | 24H MIX | 48H NT | 48H SC | 48H siRNA 1 | 48H siRNA 2 | 48H siRNA 3 | 48H MIX |
|---|---|---|---|---|---|---|---|---|---|---|---|---|
| EGFR KD% | 100 | 128.5 | 15.9 | 15.7 | 24.0 | 15.9 | 156.5 | 126.9 | 44.3 | 46.9 | 35.4 | 40.6 |
| vs. 48H NT | | | | | | | 100.0 | 81.1 | 28.3 | 30.0 | 22.6 | 25.9 |

**Supplementary Figure S6: Knockdown efficiency of siEGFR at 24 and 48 hours in the HaCaT parental cell line standardized by 24 hour no treatment (red bar) and 48 hour no treatment (blue bar).**

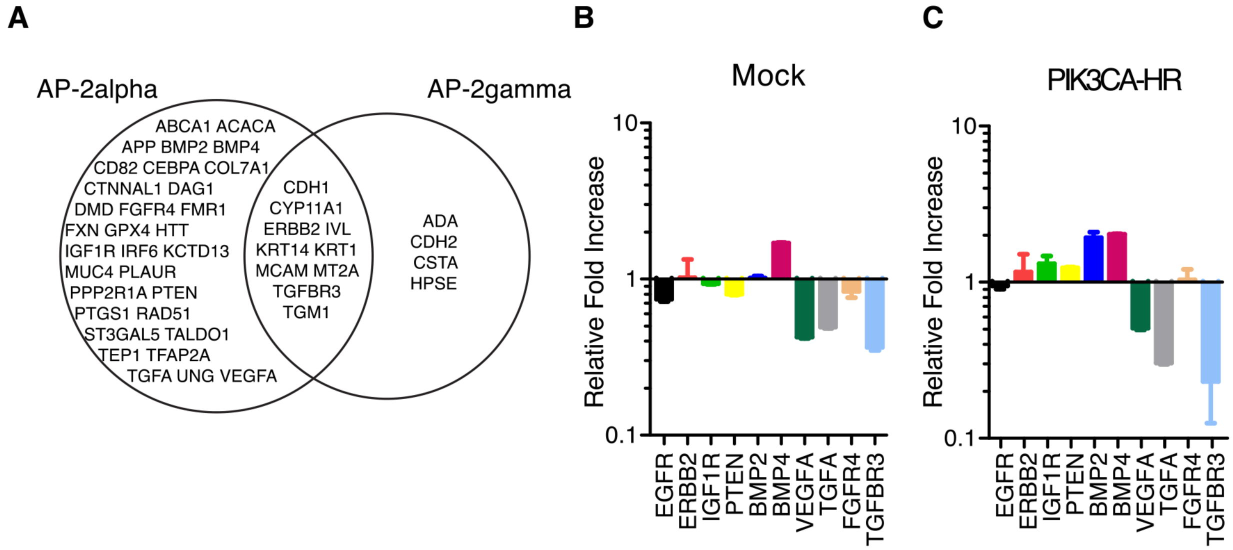


**Supplementary Figure S7: A.** Venn diagram comparing experimentally validated targets of AP-2alpha and AP-2gamma from TRANSFAC used for gene set analysis. EGFR is regulated by both TFs, but excluded from analysis to ensure enrichment is among co-regulated genes. **B.** ΔΔCt values from QRT-PCR for AP-2alpha targets after afatinib treatment relative to PBS control in HaCaT-Mock cells. **C.** ΔΔCt values from QRT-PCR for AP-2alpha targets after afatinib treatment relative to PBS in HaCaT-PIK3CA cells.

**Supplementary Table S1: Percentage of samples with mutation or copy number alterations of each gene in the EGFR network in TCGA pancreatic adenocarcinoma (PAAD), lung adenocarcinoma (LUAD), lung squamous cell carcinoma (LUSC), head and neck squamous cell carcinoma (HNSCC), and colon adenocarcinoma (COAD) samples**

| Gene | LUAD | LUSC | PAAD | COAD | HNSC |
|---|---|---|---|---|---|
| **EGFR** | 17.40% | 9.00% | 1.40% | 4.20% | 14.30% |
| **PI3K** | 17.80% | 55.10% | 11.70% | 25.90% | 41.20% |
| **RAS** | 38.70% | 8.40% | 85.50% | 50.00% | 10.80% |
| **AKT** | 12.20% | 9.60% | 13.10% | 6.10% | 8.60% |
| **RAF** | 13.90% | 6.70% | 7.60% | 11.30% | 6.80% |
| **STAT3** | 2.20% | 1.70% | 3.40% | 1.90% | 1.40% |
| **STAT5B** | 1.70% | 0.60% | 2.10% | 2.40% | 2.20% |
| **NFKB** | 7.00% | 6.20% | 6.20% | 5.70% | 6.80% |
| **FOXO** | 1.70% | 0.00% | 0.00% | 1.90% | 1.80% |
| **MEK** | 1.70% | 2.20% | 1.40% | 0.90% | 0.70% |
| **ERK** | 0.90% | 2.80% | 0.70% | 1.40% | 3.90% |
| **ELK1** | 0.90% | 0.00% | 3.40% | 0.00% | 4.70% |
| **MYC** | 10.00% | 5.10% | 14.50% | 4.20% | 13.30% |

**Supplementary Table S2: HPV-status and DNA mutations or copy number alterations for EGFR network genes in HNSCC cell lines**

| | | SCC1** [31] | SCC25* [30,31] | SQ20B* [31] | SCC61 [30,31] | SCC47 [30,31,32] | SCC90 [30,31] | 93VU147T [30,31] |
|---|---|---|---|---|---|---|---|---|
| **HPV-status** | | Negative | Negative | Negative | Negative | Positive | Positive | Positive |
| **DNA Alteration** | **EGFR** | Amplification | | | | | | |
| | **PI3K** | | | | PIK3CA p.E542K | | | |
| | **RAS** | | | | | KRAS variant rs61764370 | | HRAS p.D108Y |
| | **RAF** | | | | | | | |
| | **AKT** | | | | | | | |
| | **STAT3** | | | | NA | | | |
| | **STAT5B** | | | | NA | | | STAT5B p.P267A |
| | **NFKB** | | | | NA | | | |
| | **MEK** | | | | | | | |
| | **ERK** | | | | NA | | | |
| | **ELK1** | | | | NA | | | |
| | **MYC** | | | | NA | | | |

References for source of mutation data indicated next to each cell line name. EGFR network genes not listed in the table have no detected DNA alterations in any cell line. Table elements with NA values are missing data. * values indicate DNA alterations found from whole exome sequencing (Dataset 1 for SCC25 and Dataset 2 for SQ20B). In addition, ** values indicate DNA alterations from whole exome sequencing for SCC1 performed by the Carey and Brenner Labs (personal communication, publication in process).

**Supplementary Table S3: Cell type, origin, and DNA mutations or copy number alterations for EGFR network genes in LINCS cell lines**

| | | A375 [30] | A549 [30] | HAE1 | HCC515 [70] | HEPG2 [39] | HT29 [30] | MCF7 [30] | PC3 [30] | VCAP [30] |
|---|---|---|---|---|---|---|---|---|---|---|
| **Cell Type** | | Melanoma | Lung cancer | Immortalized normal kidney epithelial | Lung cancer | Liver carcinoma | Colon cancer | Breast cancer | Prostate cancer | Prostate cancer vertebral metastasis |
| **Source** | | ATCC | ATCC | Broad Institute | Broad Institute | ATCC | ATCC | ATCC | ATCC | ATCC |
| **DNA Alteration** | **PI3K** | | | NA | | | PIK3CA p.P449T | PIK3CA p.E545K | | |
| | **RAS** | | KRAS p.G12S | NA | | KRAS SNP, NRAS p.Q61L | | | | |
| | **RAF** | BRAF p.V600E | | NA | | | BRAF p.V600E, p.T119S | | | |
| | **STAT3** | | | NA | | | | | STAT3 loss | |
| | **STAT5B** | | | NA | | | | | STAT5B loss | |
| | **NFKB** | | | NA | NFKB2 Non-synonymous coding | | | | | |
| | **MYC** | | | NA | | | | | MYC gain | |

References for source of mutation data indicated next to each cell line name. EGFR network genes not listed in the table have no detected DNA alterations in any cell line. Table elements with NA values are missing data.

**Supplementary Table S4: Summary of LINCS scores for each therapeutic target with mean connectivity scores for the AP-2alpha gene signature across four cell lines greater than 95% (LINCS score)**

| Therapeutic | LINCS Score | A375 | A549 | HA1E | HCC515 | HEPG2 | HT29 | MCF7 | PC3 | VCAP |
|---|---|---|---|---|---|---|---|---|---|---|
| **10-DEBC** | 96.791 | 0 | 98.7 | 0 | 80.9 | 94.1 | 94.3 | 0 | 100 | -52.4 |
| **BMS-536924** | 98.0242 | 98.8 | 95.8 | 93.9 | 99.6 | 90.5 | 87.8 | 75 | -88.8 | -67 |
| **U0126** | 99.5935 | 99.9 | 96.8 | 95.2 | 99.1 | 99.4 | 94.6 | 100 | 0 | NA |
| **PD-0325901** | 99.053 | 99.7 | 95 | 99.9 | 98.5 | 97.5 | 98.1 | 83.6 | 97.2 | 96.4 |
| **PD-184352** | 98.082 | 98 | 97.9 | NA | 99.3 | 97.2 | 95.6 | 0 | 0 | -58.5 |
| **PD-0325901** | 97.4428 | 98.8 | 99.6 | 97.2 | 94.2 | 0 | 93 | 80.5 | 0 | -94.2 |
| **selumetinib** | 97.4251 | 94.4 | 90.7 | 96.2 | 99.5 | 94 | 98.2 | 95.7 | -98.2 | 65.9 |
| **U-0126** | 96.1237 | 98.4 | 0 | 77.4 | 89.3 | 98.9 | 97.9 | 76.4 | | -56.2 |
| **AZD-8055** | 98.6838 | 82.3 | 89.8 | 97.8 | 87.2 | 82.5 | 99.9 | 98.6 | 98.4 | 89.1 |
| **OSI-027** | 95.8297 | 74.6 | 69.2 | 92 | 94 | 63 | 74.9 | 96.6 | 99.7 | 93.1 |
| **tozasertib** | 95.3164 | 90.4 | 97.8 | 87.6 | 84.1 | 65.1 | 97.8 | 0 | 95.3 | -57.2 |
| **AS-605240** | 99.1907 | 99.2 | 97.9 | 74.9 | 100 | 88.6 | 99.7 | 24.2 | 0 | -46.2 |
| **wortmannin** | 97.2752 | 97 | 74.5 | 99.9 | 0 | -75.4 | 96.3 | 95.8 | 85.4 | 0 |
| **PIK-90** | 96.795 | -50.5 | 96.2 | 93.2 | 96.4 | NA | 99.3 | 87.3 | 95.2 | 72.7 |
| **PI-828** | 96.7626 | 78.6 | 66.8 | 96.4 | 99.4 | 71.8 | 97.1 | 94 | 94.2 | 85.3 |
| **tivozanib** | 96.1655 | 94.4 | 86.9 | 99.3 | NA | 78.7 | 98.4 | -22.5 | -63.4 | 92.6 |
| **sunitinib** | 97.7181 | 0 | 99.7 | 95.1 | -51.8 | 97.1 | 99 | 78.3 | -67.4 | 0 |
| **PP-110** | 98.1522 | 97.6 | 84.6 | 89.3 | 95.6 | 99.6 | 93.9 | 85.1 | 99.8 | -39.7 |
| **gefitinib** | 83.8763 | | -84.7 | 0 | 98.9 | 86.6 | 75.4 | 69.8 | 0 | 74.6 |
| **afatinib** | 81.9422 | 70.4 | 84.7 | 90.2 | 0 | 0 | -44.7 | 72.2 | 80.7 | 0 |
| **THZ-2-98-01** | -98.3237 | -98.4 | -99.8 | -51.4 | -100 | 0 | -95.1 | 0 | 70 | -44.1 |
| **MD-041** | -98.262 | -81 | -61.9 | -98.1 | -72.2 | NA | -99.1 | -99.8 | -67.8 | -96 |
| **MLN-4924** | -96.9986 | 0 | -92.8 | -96.2 | -99.4 | -98.4 | -93.9 | -59 | 0 | 0 |
| **KF-38789** | -96.7768 | -48.4 | 0 | -98.6 | 0 | -94.7 | 0 | -95.2 | -98.6 | 0 |
| **BRD-K02562327** | -95.9032 | -92.8 | NA | -99.3 | 0 | NA | -84.8 | 0 | -98.2 | -93.4 |
| **SA-247714** | -95.2425 | -94.4 | -92 | -97.8 | NA | -42.1 | 0 | -19.3 | -96.7 | -74.4 |

Connectivity values for the AP-2alpha gene signature are provided in the column corresponding to the name of each queried cell line. Doses and times used to generate the signature are available in Dataset 3.
